# Hematocrit and the Risk of Recurrent Venous Thrombosis: A Prospective Cohort Study

**DOI:** 10.1371/journal.pone.0038705

**Published:** 2012-06-06

**Authors:** Lisbeth Eischer, Verena Tscholl, Georg Heinze, Ludwig Traby, Paul A. Kyrle, Sabine Eichinger

**Affiliations:** 1 Department of Medicine I, Medical University of Vienna, Vienna, Austria; 2 Core Unit for Medical Statistics and Informatics, Medical University of Vienna, Vienna, Austria; Leiden University Medical Center, The Netherlands

## Abstract

**Background:**

Venous thromboembolism (VTE) is a multicausal disease which recurs. Hematocrit is associated with a thrombotic risk. We aimed to investigate if hematocrit is associated with the recurrence risk.

**Methods:**

Patients with a first VTE were followed after anticoagulation. Patients with VTE provoked by a transient risk factor, natural inhibitor deficiency, lupus anticoagulant, homozygous or double heterozygous defects, cancer, or long-term antithrombotic treatment were excluded. The study endpoint was recurrent VTE.

**Results:**

150 (23%) of 653 patients had recurrence. Only high hematocrit was significantly associated with recurrence risk [hazard ratio (HR) for 1% hematocrit increase with the third tertile 1.08; 95% CI 1.01–1.15]. No or only a weak association for hematocrits within the first and second tertile was seen (HR 1.03; 95% CI 0.97–1.09, and 1.07; 95% CI 1.00–1.13). Hematocrit was associated with recurrence risk only among women. After five years, the probability of recurrence was 9.9% (95% CI 3.7%–15.7%), 15.6% (95% CI 9.7%–21.2%) and 25.5% (95% CI 15.1%–34.6%) in women, and was 29.2% (95% CI 21.1%–36.5%), 30.1% (95% CI 24.1%–35.7%) and 30.8% (95% CI 22.0%–38.7%) in men for hematocrits in the first, second and third tertile, respectively. Men had a higher recurrence risk (1.9; 95% CI 1.1–2.7; p = 0.03), which dropped by 23.5% after adjustment for hematocrit. Hematocrit was not a significant mediator of the sex-difference in recurrence risk (p = 0.223).

**Conclusions:**

High hematocrit is associated with the recurrence only in women. The different recurrence risk between men and women is possibly partly explained by hematocrit.

## Introduction

Venous thromboembolism (VTE) is a multicausal disease, which tends to recur. The risk of recurrence depends on the severity and number of risk factors that are present in a patient at a certain time point. Meanwhile, the impact of many acquired and genetic risk factors on the recurrence risk has been defined [1], and prediction models that integrate these factors have been developed [2], [3]. With regard to clinical relevance, the patient's sex is one of the most important factors, as men have a higher recurrence risk than women [4]. Overall, the sex-related risk increase is about 2-fold [5], is present in all age groups, is not related to hormone contraceptive intake at the first thrombotic event, and independent also of other established risk factors [6].

Men and women differ with regard to hematocrit levels. The difference is most pronounced between premenopausal women and age-matched men [7]. Hematocrit correlates with the thrombotic risk. High hematocrit is associated with increased plasma viscosity and platelet reactivity [8], [9]. Patients with myeloproliferative disorders, particularly those with polycythemia vera, are at high risk of thrombosis [10], [11]. In a case control study the percentage of study participants with a hematocrit above 45% was higher in DVT patients (43%) than in healthy controls (27%) [12]. High hematocrit was a risk factor of a first VTE in the general population [13].

We hypothesized that hematocrit may be causally related to the recurrence risk, which may then provide an explanation for the risk difference between men and women. The aim of our study was to investigate the association between hematocrit and the risk of recurrent VTE and to evaluate the effect of hematocrit on the recurrence risk in men and women.

## Methods

### Patients and study design

This study was performed within the frame of the Austrian Study on Recurrent Venous Thromboembolism (AUREC), a large prospective ongoing multi-centre cohort study initiated in 1992. Recruitment of new patients was stopped in September 2008. The present analysis considers follow-up data collected until October 2009. Patients were included, when they were older than 18 years of age, had a symptomatic objectively confirmed VTE, and were treated with anticoagulants for 3 to 18 months. Exclusion criteria were a previous episode of VTE; VTE related to surgery, trauma, pregnancy or female hormone intake; upper extremity DVT; deficiency of antithrombin, protein C, or protein S, presence of the lupus anticoagulant or homozygosity or double heterozygosity for factor V Leiden and/or factor II G20210A; cancer at time of enrolment; or requirement for long-term antithrombotic treatment for other reasons than VTE. Patients entered the study at the time of discontinuation of anticoagulation. At study entry, a detailed medical history was obtained, and a physical examination and body mass index (BMI) calculation were performed. According to their smoking habits at time of index-VTE patients were categorized as smokers or non-smokers. Three weeks after withdrawal of anticoagulation, they were screened for deficiency of antithrombin, protein C, or protein S, the lupus anticoagulant, factor V Leiden and factor II G20210A and a complete blood count was performed. Patients were seen at six-month intervals for the first year and once a year thereafter. They were given detailed written information on symptoms of VTE and were asked to report immediately if such symptoms occurred. Female patients were strongly discouraged from intake of estrogen-containing oral contraceptives or hormone replacement therapy. Patients received thromboprophylaxis with a low-molecular-weight heparin during high-risk situations such as surgery, trauma, prolonged immobilization or long-haul air travel. The ethics committee of the Medical University of Vienna approved the study and all patients gave written informed consent prior to inclusion in the study.

### Diagnosis of first venous thromboembolism

As previously described in more detail [5], the diagnosis of VTE was established by a positive finding on venography or color duplex sonography.

The diagnosis of PE was established by ventilation-perfusion lung scanning or by spiral computed tomography (CT) scan.

### Study end points

The end point of the study was recurrent symptomatic DVT confirmed by venography or color duplex sonography, or recurrent symptomatic PE confirmed by multi-slice computed tomography, ventilation-perfusion lung scanning or autopsy.

### Laboratory analysis

Venous blood from fasting patients was collected into EDTA tubes or in 1∶10 volume of 0.11 mM trisodium citrate and immediately centrifuged for 20 min at 2000×*g*. Aliquots of plasma were stored at −80°C until analysis.

Complete blood count including hematocrit was determined in EDTA blood using a Sysmex XE-2100-analyser.

Screening for factor V Leiden and prothrombin G20210A and measurement of antithrombin, protein C and protein S were carried out by standard methods. The diagnosis of the lupus anticoagulant was based on criteria of the International Society on Thrombosis and Haemostasis [14].

### Statistical analysis

Categorical data were compared among groups using contingency-table analyses (the chi-square test). Continuous data (presented as means ± SD) were compared by means of Mann-Whitney U tests. Survival-time methods were used to analyse the time to recurrence among patients with a subsequent episode of VTE (uncensored observation) or the duration of follow-up among patients without recurrence (censored observations) [15]. Data on patients who left the study or who were lost to follow-up were censored at the time of withdrawal. The probability of recurrence was estimated according to the method of Kaplan and Meier [16]. To test for homogeneity between strata, we applied the log-rank test. Univariate and multivariate Cox proportional-hazards model were used to analyse the association between hematocrit and the risk of recurrent VTE.

To assess the effect of hematocrit and sex on the recurrence risk and to investigate linearity or non-linearity in case of an association, we accounted for a possibly non-linear effect of hematocrit by non-linear estimation via fractional polynomials. The fractional polynomial (FP) technique is extensively described elsewhere [17], here we just briefly describe its outline. Instead of using hematocrit as linear in the Cox regression, this technique evaluates several pre-specified transformations of hematocrit, and selects that transformation or combination of transformations that leads to the best-fitting model. After determining the best transformation of hematocrit, product terms of sex and the transformed hematocrit levels were included in the model to evaluate and test the interaction of sex and hematocrit. The final nonlinear interaction model was used to estimate the cumulative recurrence rates of women having hematocrits of 36%, 40% and 43%, corresponding to the tertile means of women, and of men having hematocrits of 41%, 44% and 47%, corresponding to the tertile means in men. In these computations, average covariate values were inserted for all other variables (age, BMI, location of initial VTE, smoking status, factor V Leiden).

Mediation of the sex effect by hematocrit was evaluated by comparing the hematocrit-adjusted and -unadjusted log hazard ratios of sex in the Cox regression model. A P-value for the null hypothesis that the hematocrit-adjusted and –unadjusted log hazard ratios of sex are equal (no mediation) was obtained using the method of Lin et al [18]. All P values were two-tailed, and were considered as indicating statistical significance if lower than 0.05. The statistical software R 2.9.2 (R development core team, Vienna, 2009, www.r-project.org) and the R package coxphf (written by G. H.), which combines the fractional polynomial technique with a small-sample bias-correction method in Cox regression, was used for statistical analysis [19]. In addition, SPSS software, version 15.0, was used.

## Results

### Study population

A total of 653 patients, 427 (65%) men and 226 (35%) women, with a first unprovoked DVT and/or PE were followed for a mean of 43 months(Table 1).171 patients left the study because of newly diagnosed cancer (17), pregnancy (5), antithrombotic therapy for reasons other than VTE (94) or loss to follow-up (43). 12 patients died for reasons other than VTE. Patients were followed until the time of exclusion or death, when data were censored.

**Table 1:** Patient characteristics.

		Total cohort	Women	Men	*p* for sex difference
		(n = 653)	(n = 226)	(n = 427)	
Age (yrs)		52 ± 14	55 ± 15	51 ± 14	0.03
Location of first VTE	DVT distal – n(%)	110 (17)	46 (20)	64 (15)	
	DVT proximal – n(%)	254 (39)	70 (31)	184 (43)	0.01
	PE ± DVT – n(%)	289 (44)	110 (49)	179 (42)	
Duration of anticoagulation (mo)		7.5 ± 2.4	7.6 ± 2.6	7.5 ± 2.3	0.6
Observation time (mo)		43 ± 33	42 ± 35	43 ± 32	0.5
Factor V Leiden – n(%)		154 (24)	40 (18)	114 (27)	0.01
Factor II G20210A – n(%)		30 (5)	12 (5)	18 (4)	0.6
Body mass index (kg/m^2^)		27.8 ± 5.0	28.2 ± 6.1	27.6 ± 4.3	0.5
Smoking status – n(%)		186 (30)	60 (28)	126 (31)	0.4
Hematocrit (%)		42.3 ± 3.6	39.7 ± 3.3	43.7 ± 2.9	<0.001

Plus-minus values are means ± standard deviation.

### Recurrence of venous thromboembolism

Symptomatic VTE recurred in 150 (23%) patients. Isolated DVT occurred in 77 and PE with or without DVT in 73 patients, 3 of them were fatal. Male sex conferred a hazard ratio (HR) of recurrence of 1.9 (95% CI 1.3–2.9; p = 0.001). The univariate HR for age (per decade), BMI, smoking status, factor V Leiden and factor II G20210A were 0.97 (95% CI 0.86–1.09, p = 0.63), 1.04 (95% CI 1.0–1.07, p = 0.03), 0.98 (95% CI 0.69–1.41, p = 0.93), 1.67 (95% CI 1.19–2.35, p = 0.003), and 1.05 (95% CI 0.52–2.14, p = 0.89), respectively.

### Hematocrit and risk of recurrent VTE

In a Cox proportional hazards model, the HR of recurrence was 1.07 (95% CI 1.02–1.13; p = 0.004) for each 1% increase in hematocrit. In a nonlinear modeling using the technique of fractional polynomials, the HR related to a 1% increase in hematocrit was not constant over the range of hematocrit values, but depended on whether hematocrit was low or high. While in a multivariable model (adjusted for sex, age, BMI, location of first VTE, smoking status and factor V Leiden) no association between a 1% increase of hematocrit within the lowest tertile (mean value 38.5%) was seen, the association was stronger for hematocrits in the second tertile (mean value 42.5%) and was significant in the highest tertile (mean value 46.0%): HR 1.03 (95% CI 0.97–1.09), 1.07 (95% CI 1.00–1.13) and 1.08 (95% CI 1.01–1.15), respectively.

#### Recurrence risk in women and men according to hematocrit

**Figure 1:**
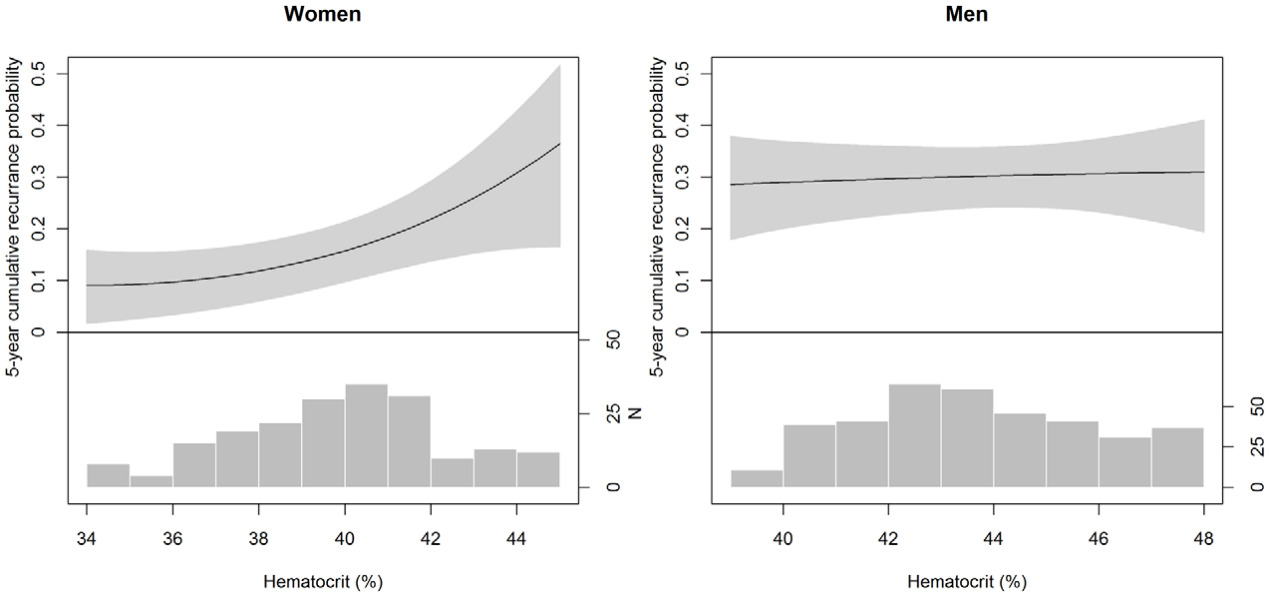
Five-year cumulative recurrence risk as estimated from the Cox regression model in women (left) and men (right) at various hematocrits, adjusted for location of first venous thromboembolism, body mass index, age, factor V Leiden, and smoking status. The gray-shaded area corresponds to the 95% confidence intervals. The histograms at the bottom show the frequency distribution (n = number of patients) of hematocrit in women and men.

Hematocrit was significantly higher in men than in women (43.7% vs. 39.7%, p<0.001), and the frequency distribution of hematocrit differed substantially (Figure 1). We therefore hypothesized that the effect of sex on recurrence risk might be explained by the sex-specific hematocrit levels and the relationship between hematocrit and risk of recurrence.

VTE recurred in 32 of the 226 women (14.2%) and in 118 of the 427 men (27.6%). Male sex remained a significant predictor of the recurrence risk after adjustment for hematocrit [HR 1.7 (95% CI 1.1–2.7), p = 0.03] using linear or non-linear values. Although the HR of recurrence for male sex dropped from 1.9 to 1.7 after adjustment for hematocrit, the formal test for mediation did not reach significance (p = 0.223).

**Figure 2:**
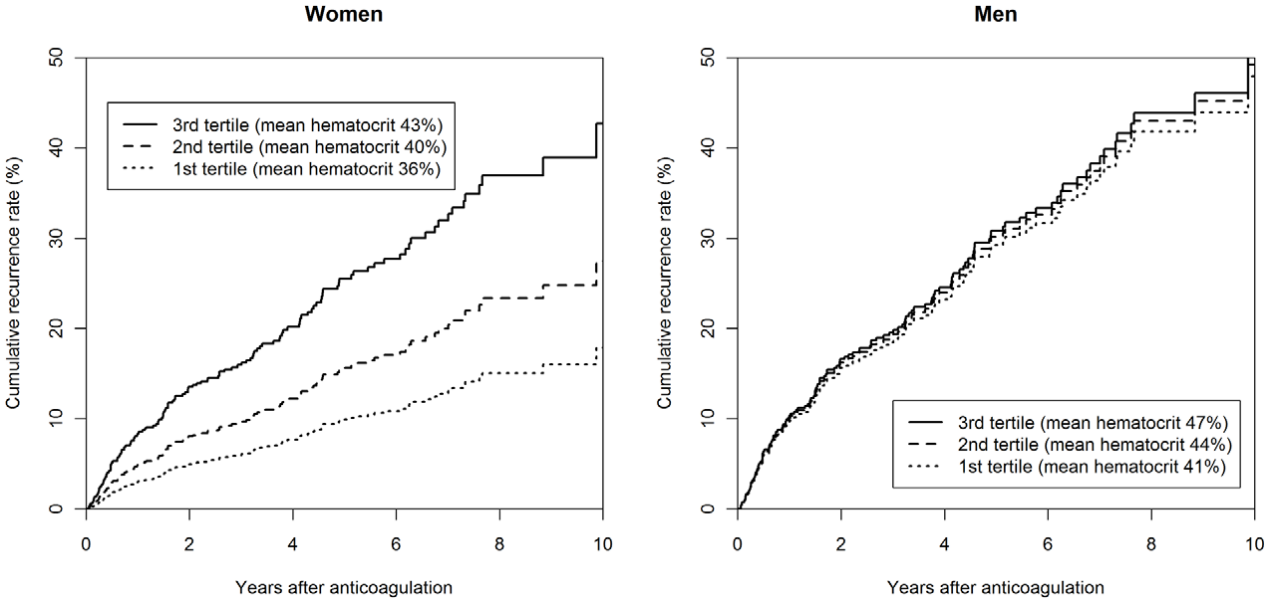
Cumulative recurrence rates (as estimated from the Cox regression model) in women and men according to tertile mean values of hematocrit, adjusted for location of first venous thromboembolism, body mass index, age, factor V Leiden, and smoking status.

We further investigated whether the effect of hematocrit on recurrence risk differed between women and men using interaction analysis. Here, product terms of the transformed hematocrit values with sex (H_1_×sex and H_2_×sex) were introduced to the model. If different from zero, these product terms indicate a sex-related difference in the association between hematocrit and the risk of recurrence. Indeed, the two terms were simultaneously significantly different from zero (p = 0.024). This implied that the risk of recurrence associated with a 1% increase in hematocrit not only depended on the level of hematocrit but also on the sex of the patients. In order to illustrate this nonlinear sex-dependent effect of hematocrit, we computed the HRs at the sex-specific hematocrit tertile mean values (36.3%, 40.0% and 42.9% in women; 40.7%, 43.6% and 46.8% in men) (Table 2). While hematocrit had no relevant impact on the recurrence risk in men, its effect was strong and nonlinear in women. From this final Cox regression model we also derived the five-year cumulative recurrence rates in women and in men (Figure 1). Furthermore, we estimated and depicted the cumulative recurrence risk for women with hematocrits of 36%, 40% and 43% (corresponding to the tertile means of female patients), and for men of 41%, 44% and 47% (corresponding to the tertile means of men) (Figure 2). After five years, the probability of recurrence was 9.9% (95% CI 3.7%–15.7%), 15.6% (95% CI 9.7%–21.2%), and 25.5% (95% CI 15.1%–34.6%) in women with hematocrit ranging in the lowest to the highest tertile and was 29.2% (95% CI 21.1%–36.5%), 30.1% (95% CI 24.1%–35.7%), and 30.8% (95% CI 22.0%–38.7%) in men within the respective tertiles.

**Table 2:** Risk of recurrent venous thromboembolism accounting for the nonlinear interaction of hematocrit* and sex†.

	Male patients		Female patients	
Tertile	Mean hematocrit (%)	Hazard ratio‡	Mean hematocrit (%)	Hazard ratio‡
		/1% increase in hematocrit (95% CI)		/1% increase in hematocrit (95% CI)
1	40.7	1.01 (0.93, 1.10)	36.3	1.11 (0.99, 1.23)
2	43.6	1.01 (0.93, 1.09)	40.0	1.19 (1.06, 1.34)
3	46.8	1.01 (0.91, 1.12)	42.9	(1.09, 1.38)

* hematocrit (H) was modelled using (H/100)−2 and (H/100)−2log(H/100).

† the *p*-value for the interaction product terms sex*(H/100)−2 and sex*(H/100)−2log(H/100) was 0.024 (2 degrees of freedom).

‡ adjusted for age, body mass index, smoking status and factor V Leiden.

## Discussion

Our study shows that hematocrit is associated with the risk of recurrence in patients with a first unprovoked VTE. Herewith, we provide further clinical evidence that corroborates the relation between hematocrit and the risk of VTE [11], [12]. We considered several factors that might affect hematocrit and could therefore confound our results. We excluded patients with cancer and pregnancy related thrombosis as well as those who required anticoagulant treatment because of an underlying heart condition. Hematocrit was determined after discontinuation of anticoagulation, i.e. at least three months after VTE, which precludes an effect of the acute thrombotic event on hematocrit. We adjusted our analyses for age, BMI, smoking status, and factor V Leiden, but the association between hematocrit and recurrence risk was independent of these potential confounders. Nevertheless, whether or not this relation is causal remains open.

Notably, the association between hematocrit and recurrence risk was dependent on the level of hematocrit and the sex of the patient. First, the relation between hematocrit and recurrence risk was not constant. While low hematocrits (values within the first tertile) were not associated with the recurrence risk, hematocrits within the highest tertile were strongly predictive. Second, hematocrit was useful to stratify patients according to recurrence risk only in women. Women with hematocrit less than 36% (corresponding to the upper limit of the first tertile) had a very low probability of recurrence corresponding to 9.9% (95% CI 3.7%–15.7%) after five years. In men, no association between hematocrit and recurrence risk was found. These findings are in contrast to the study by Braekkan, where the association between hematocrit and risk of thrombosis was more pronounced in men than in women [12]. In that study the relationship between hematocrit and a first rather than recurrent VTE was investigated. While we included only patients with unprovoked events, in the study by Braekkan more than 60% had provoked VTE, the proportion of women who had provoked events was higher than that of men and data of women who had their VTE during female hormone intake or pregnancy were also included.

In our study, the risk of recurrence was 1.9-fold higher in men than in women. This is lower than what we previously published [4], but is due to exclusion of women who had VTE during female hormone intake in the present analysis.

Hematocrit in average was higher in men than in women (43.7% vs. 39.7%). Nevertheless, our hypothesis that the sex-difference in recurrence rates could be explained by hematocrit was only partly supported by our analyses. When we adjusted the regression analysis of the recurrence risk between men and women for hematocrit, the risk was still significantly higher among men but the HR was reduced to 1.7. However, after formal testing this risk difference was not significant, which indicates that hematocrit, if at all, is only a minor mediator of the sex effect on recurrence risk. Some strengths and limitations of the current study have to be addressed. Our results are based on a large study population with a long observation time. An objective diagnosis and end point verification was established in all patients. Hematocrit was measured only once shortly after discontinuation of anticoagulation. We therefore could not account for variations in hematocrit over time and a potential association on the risk of recurrent VTE. Potential confounders of the hematocrit including pack years of smoking, lung disease, kidney function, diuretic use or chronic inflammatory disease were not collected systematically. We did not evaluate the association between other variables of red blood cell characteristics. Our findings cannot be extrapolated to patients with a provoked VTE or those with a strong thrombophilic defect, as these patients were excluded. The Austrian Study on recurrent Venous Thromboembolism is a hypothesis-generating cohort study, which precludes predefinition of certain cutoff values.

In summary, we found a nonlinear and sex-dependent relationship between hematocrit and risk of recurrent VTE among patients with a first unprovoked venous thrombosis. Women with low hematocrit are at low risk of recurrence. Hematocrit is not predictive for recurrence in men. The difference in the risk of recurrent VTE between men and women is possibly partly explained by the sex-related difference in hematocrit.

## References

[1] Kyrle PA, Rosendaal FR, Eichinger S (2010). Risk assessment of recurrent venous thrombosis.. Lancet.

[2] Rodger MA, Kahn SR, Wells PS, Anderson DA, Chagnon I (2008). Identifying unprovoked thromboembolism patients at low risk for recurrence who can discontinue anticoagulant therapy.. CMAJ.

[3] Eichinger S, Heinze G, Jandeck LM, Kyrle PA (2008). Risk assessment of recurrence in patients with unprovoked deep vein thrombosis or pulmonary embolism: the Vienna prediction model.. Circulation.

[4] Kyrle PA, Minar E, Bialonczyk C, Hirschl M, Weltermann A (2004). The risk of recurrent venous thromboembolism in men and women.. N Engl J Med.

[5] McRae S, Tran H, Schulman S, Ginsberg J, Kearon C (2006). Effect of patient's sex on risk of recurrent venous thromboembolism: a meta-analysis.. Lancet.

[6] Douketis J, Tosetto A, Marcucci M, Baglin T, Cosmi B (2011). Risk of recurrence after venous thromboembolism in men and women: patient level meta-analysis.. BMJ.

[7] Bloom AL CDF, Duncan TP, Tuddenham (1994). EGD: Haemostasis and thrombosis.. Longman Group UK; Churchill Livingstone Edinburgh London Madrid Melbourne New York And Tokyo.

[8] Santos MT, Valles J, Marcus AJ, Safier LB, Broekman MJ (1991). Enhancement of platelet reactivity and modulation of eicosanoid production by intact erythrocytes. A new approach to platelet activation and recruitment.. J Clin Invest.

[9] Valles J, Santos MT, Aznar J, Marcus AJ, Martinez-Sales V (1991). Erythrocytes metabolically enhance collagen-induced platelet responsiveness via increased thromboxane production, adenosine diphosphate release, and recruitment.. Blood.

[10] Gruppo Italiano Studio Policitemia (1995). Polycythemia vera: the natural history of 1213 patients followed for 20 years.. Ann Intern Med.

[11] Schafer AI (1984). Bleeding and thrombosis in the myeloproliferative disorders.. Blood.

[12] Vayá A, Mira Y, Martínez M, Villa P, Ferrando F (2002). Biological risk factors for deep vein thrombosis.. Clin Hemorheol Microcirc.

[13] Braekkan SK, Mathiesen EB, Njølstad I, Wilsgaard T, Hansen JB (2010). Hematocrit and risk of venous thromboembolism in a general population. The Tromso study.. Haematologica.

[14] Brandt JT, Triplett DA, Alving B, Scharrer I (1995). Criteria for the diagnosis of lupus anticoagulants: an update.. Thromb Haemost.

[15] Kalbfleisch JD, Prentice RL (1980). The Statistical Analysis of Failure Time Data (Wiley Series in Probability and Statistics)..

[16] Kaplan EL, Meier P (1958). Nonparametric estimation from incomplete observations.. J Am Stat Assoc.

[17] Royston P, Sauerbrei W (2008). Multivariable Model-building – A pragmatic approach to regression analysis based on fractional polynomials for modelling continuous variables..

[18] Lin DY, Fleming TR, De Gruttola V (1997). Estimating the proportion of treatment effect explained by a surrogate marker.. Statistics in Medicine.

[19] Heinze G, Schemper M (2001). A solution to the problem of monotone likelihood in Cox regression.. Biometrics.

